# Functional profile of patients with behavioral variant frontotemporal dementia (bvFTD) compared to patients with Alzheimer's disease and normal controls

**DOI:** 10.1590/S1980-57642013DN70100015

**Published:** 2013

**Authors:** Thais Bento Lima-Silva, Valéria Santoro Bahia, Viviane Amaral Carvalho, Henrique Cerqueira Guimarães, Paulo Caramelli, Márcio Balthazar, Benito Damasceno, Cássio Machado de Campos Bottino, Sônia Maria Dozzi Brucki, Ricardo Nitrini, Mônica Sanches Yassuda

**Affiliations:** 1 Neurology Department, University of São Paulo, São Paulo SP, Brazil.; 2 Behavioral and Cognitive Neurology Unit, Department of Internal Medicine, Federal University of Minas Gerais, Belo Horizonte MG, Brazil.; 3 Department of Neurology, University of Campinas, Campinas SP, Brazil.; 4 Institute of Psychiatry, University of São Paulo, São Paulo SP, Brazil.

**Keywords:** behavioral variant frontotemporal dementia, functional status, dependence

## Abstract

There are few studies describing the functional changes in behavioral variant frontotemporal dementia (bvFTD) and it is not clear which aspects of functionality are affected by the disease.

**OBJECTIVE:**

The aim of the present investigation was to characterize the functional profile of patients previously diagnosed with bvFTD.

**METHODS:**

The sample consisted of 31 patients diagnosed with bvFTD, who were compared to patients with Alzheimer's disease (AD) (n=31) and to healthy control subjects (NC) (n=34), matched for schooling and age. bvFTD and AD patients were matched by severity of dementia. The protocol included the Mini-Mental State Examination (MMSE), Geriatric Depression Scale (GDS), Direct Assessment of Functional Status (DAFS-BR), Functional Activities Questionnaire (PFAQ), Disability Assessment for Dementia (DAD) and the Clinical Dementia Rating scale (CDR).

**RESULTS:**

The group with bvFTD showed worse performance on Initiation and Planning/Organization in the DAD and on ability to feed oneself in the DAFS-BR, as well as higher scores on the PFAQ, suggesting greater dependence in the bvFTD group.

**CONCLUSION:**

The results suggest that individuals with bvFTD display greater functional impairment compared to AD patients with a similar degree of dementia severity and to healthy controls. Direct assessment of functionality proved unable to clearly differentiate between the dementia subtypes.

## Abstract (pt)

Existem poucos estudos sobre alterações funcionais na variante comportamental da demência frontotemporal (DFTvc).

**OBJETIVO:**

Caracterizar o desempenho funcional de pacientes com diagnóstico prévio de DFTvc.

**MÉTODOS:**

Trinta e um pacientes com DFTvc foram comparados a pacientes com doença de Alzheimer (DA) (n=31) e adultos saudáveis (NC) (n=34), pareados para idade e escolaridade. Os pacientes com DFTvc e DA foram pareados pela gravidade da demência. O protocolo incluiu o Mini Exame do Estado Mental, Escala de Depressão Geriátrica (GDS), *Direct Assessment of Functional Status* (DAFS-BR), *Disability Assessment for Dementia* (DAD), *Functional Activities Questionnaire* (PFAQ) e *Clinical Dementia Rating scale* (CDR).

**RESULTADOS:**

O grupo com DFTvc apresentou pior desempenho em Iniciação e Planejamento/Organização na DAD, em Alimentação na DAFS-BR e pontuação mais elevada na PFAQ, sugerindo que a dependência na DFTvc é mais acentuada.

**CONCLUSÃO:**

Os resultados apresentados sugerem que indivíduos com DFTvc apresentam maior prejuízo funcional, quando comparados com participantes com DA com grau semelhante de gravidade e com adultos saudáveis. A avaliação direta da funcionalidade não ajudou a diferenciar os subtipos de demência de modo significativo.

## INTRODUCTION

Behavioral variant frontotemporal dementia (bvFTD) is a clinical syndrome characterized by progressive impairment in behavior, personality, as well as social, cognitive and functional abilities, which predominantly affects middle-aged adults.^1,2^ Despite recent advances in characterizing bvFTD, diagnosing this syndrome remains challenging. While some patients are erroneously deemed cognitively preserved, others are diagnosed with psychiatric disorders or Alzheimer's disease (AD).^3^

In 2011, a set of revised diagnostic criteria was proposed for the bvFTD. With the revised criteria, a diagnosis of "possible" bvFTD requires three of the six clinically discriminated characteristics: loss of inhibition, apathy/inertia, loss of empathy, perseveration/compulsive behaviors, hyperorality and dysexecutive neuropsychological profile. "Probable" bvFTD requires the additional features of functional disability and characteristic neuroimaging, whereas bvFTD "with definitive frontotemporal lobar degeneration" requires histopathological confirmation or evidence of pathogenic mutation. Therefore, investigating functionality is essential for reaching the diagnosis and also relevant for the treatment of the syndrome, given that the impact on the activities of daily living can be used as a clinical parameter.^4-7^ However, studies investigating the functional performance of patients with bvFTD and other subtypes of frontotemporal lobar degeneration, such as non-fluent progressive aphasia (NFPA) and semantic dementia (SD), are scarce.^8,9^

Using the Disability Assessment for Dementia (DAD) questionnaire, Mioshi et al.^9^ showed that patients with bvFTD had poorer functional performance than patients with SD, NFPA or AD. The bvFTD group had lower scores even on basic activities of daily living (BADLs), such as getting dressed, feeding and hygiene. For the instrumental activities of daily living (IADLs), the worst performances were seen in finances, correspondence and going on an outing. Compared to AD patients, poorer performances were also evident for the use of the telephone, domestic and leisure time activities, managing medications, and meal preparation. The authors highlighted the devastating impact of functional changes on the everyday routine of patients with bvFTD and the burden placed on their caregivers. These results were confirmed in a later study by Kipps et al.^10^

In another later study, Mioshi et al.^11^ sought to examine the rate of changes in activities of daily living (ADL). The patients were subdivided into bvFTD pathological and phenocopy subgroups, SD and PNFA. The results indicated that pathological bvFTD, SD and PNFA groups showed significant decline in ADL after 12 months, while the phenocopy subgroup did not. Patients with SD declined at a slower pace, similar to that reported in AD studies. Functional and cognitive scores were significantly correlated. In agreement with these findings, Josephs et al.^1^ reported that poor performance on executive, visuospatial and language functions were indicative of more rapid decline in functional activities.

A study by Wicklund et al.^12^ examined the functional profile of patients diagnosed with AD, bvFTD and primary progressive aphasia (PPA). Results showed that functional ability was moderately impaired in AD and bvFTD, and mildly impaired in PPA. Self-care activities were the least impaired in all groups, whereas more complex ADLs, such as shopping and management of finances, were impaired early on. Communication ability was the least impaired, along with self-care for bvFTD and AD, and the most impaired for PPA patients.

Although scarce, previous studies have suggested that bvFTD patients may exhibit a more marked rate of functional decline. In addition, the profile of functional impairment may also differ. However, previous studies comparing different dementia sub-types have relied solely on indirect measures of performance, based on the informants' perceptions of the functional abilities of patients. Therefore, the aim of the present investigation was to characterize the functional profile of bvFTD patients, based on direct and indirect functional performance measures, compared to patients with AD and normal controls (NC).

## METHODS

**Participants and procedures.** Individuals with bvFTD and AD and their caregivers were invited to participate from existing case series at the following institutions: Cognitive and Behavioral Neurology Group (GNCC-SP) at the Department of Neurology, School of Medicine - University of São Paulo (FMUSP); Program for the Elderly (PROTER) at the Institute of Psychiatry, FMUSP; Cognitive and Behavioral Neurology Group (GNCC-MG) at the Department of Internal Medicine, Faculty of Medicine - Federal University of Minas Gerais; and the Department of Neurology, School of Medical Sciences, State University of Campinas (UNICAMP). The individuals in the control group (CG) were recruited from participants at a University of the Third Age at the School of Arts, Sciences and Humanities (EACH), University of São Paulo.

Ninety-six individuals, aged 55 or older and with at least two years of formal education, were invited to participate. Thirty-one had been previously diagnosed with bvFTD and 31 with AD. bvFTD and AD patients were matched according to the severity of the disease, based on the Clinical Dementia Rating (CDR) scores. All patients had a family member or caregiver who could complete questionnaires during the interviews. Additionally, 34 healthy adults, matched to patients with bvFTD and AD for age and education, were recruited.

Patients with dementia were previously diagnosed by the neurologists or psychiatrists from the above-mentioned research centers, who based their diagnosis on clinical and cognitive assessments, laboratory tests and on neuroimaging. For the bvFTD diagnosis, the criteria by Neary et al.^13^ were used. Dementia was diagnosed according to the DSM-IV criteria^14^ and AD diagnosis followed the NINCDS-ADRDA criteria.^15^

The following individuals were excluded from the sample: patients aged 45 or younger; individuals with visual, hearing or motor impairments which hindered comprehension of instructions and execution of cognitive tasks; individuals with other uncontrolled conditions such as hypertension and diabetes; individuals with psychiatric disorders such as severe depression, bipolar disorder, and schizophrenia; individuals with clinical evidence or neuroimaging exams pointing to severe vascular impairment; individuals with other types of dementia.

Regarding the control group, individuals with Geriatric Depression Scale (GDS) scores of six or higher^16,17^ and Mini-Mental State Examination (MMSE)^18^ scores below the cutoff point for cognitive impairment, were excluded. The following education adjusted cutoff points were used: illiterate, 17 points; 1 to 4 years of schooling, 22 points; 5 to 8 years of schooling, 24 points; more than 8 years of schooling, 26 points. These cutoff points have been adapted from Brucki et al.,^18^ considering the means for each level of schooling minus one standard deviation.

The protocol proposed by the present research study was implemented within the outpatient clinic of each of the institutions concerned, in a room reserved for this purpose, with adequate lighting and noise levels. The administration of the protocol took about 60 minutes among patients and about 45 minutes among healthy adults. The interview with informants lasted around 45 minutes.

**Instruments.** The following instruments were applied to patients and controls: sociodemographic and clinical questionnaire; the Mini-Mental State Examination (MMSE);^18^ Geriatric Depression Scale (GDS) with 15 items^16,17^ and Direct Assessment of Functional Status (DAFS-BR).^19,20^

The protocol for caregivers included the following instruments: Pfeffer Functional Activities Questionnaire (PFAQ);^21^ Disability Assessment for Dementia (DAD)^22,23^ and Clinical Dementia Rating scale (CDR).^24-26^

**Sociodemographic and clinical characteristics.** The sociodemographic and clinical variables assessed included age, income, years of schooling, marital status, overall health and wellness, presence of other clinical conditions, and the use of pharmacological drugs. This component of the protocol was applied to controls and to caregivers of patients with dementia.

**Cognitive and neuropsychiatric instruments.** The MMSE is the most frequently used cognitive screening test and assesses several cognitive domains. Score ranges from 0 to 30 points, with higher scores indicating better cognitive performance.

The GDS is one of the most widely used instruments to screen for depression among older adults. The GDS-15 has been reported to have adequate psychometric characteristics when used in the Brazilian elderly population.^16,17^

The CDR was devised to assess the severity of dementia, in particular in AD.^24,25^ This scale includes the assessment of memory, orientation, judgment and problem solving, community affairs, home and hobbies, and personal care. Part of the assessment is conducted with the patient and an additional semi-structured interview is conducted with the caregiver. After data collection, the clinician makes an appraisal of each domain and an overall appraisal of the cognitive status of the patient, assigning scores of 0, 0.5, 1, 2 or 3. The CDR has been shown to have good reliability as a tool for categorizing the severity of AD.^26^

**Functional performance.** The DAFS-BR measures functional performance based on the observation of the patient's performance while he/she carries out activities of daily living. It comprises six sub-tests, such as making a phone call, simulating grocery shopping, recognizing bills and coins, checking the change in a transaction, balancing a checkbook, performing self-care activities, among others.^19,20^ The DAFS-BR consists of the following sub-domains: time orientation (score range 0-16), communication (score range 0-15), ability to handle money (score range 0-32), ability to shop (score range 0-20), ability to get dressed (score range 0-10) and ability to feed oneself (score range 0-13). Score ranges from 0 to 106.

The PFAQ is a functional assessment instrument based on the informant's perception of the patient's functional ability. It consists of ten items that examine the degree of independence when the patient performs activities of daily living. The score ranges from 0 to 30, and the higher the score, the greater the degree of dependence of the patient.^21^

The DAD is used to assess functional impairment in dementia based on a caregiver's report. It includes BADLs, such as getting dressed, hygiene and nutrition,^22^ as well as IADLs, such as preparing meals, using the phone, doing housework, dealing with the mail and one's finances, enjoying recreational activities, managing medication, and being able to safely stay at home. The DAD is organized according to the essential components of the tasks: initiation, planning and organization, and effective performance. Score ranges from 0 to 100 and higher scores indicate better performance.^23^

**Ethical aspects.** The present study was approved by the Ethics Committee for Evaluation of Research Projects (CAPPesq) of the Hospital das Clínicas, School of Medicine, University of São Paulo, protocol number 0457/10. NC participants and caregivers of patients with dementia filled out the informed consent form and were instructed about research procedures.

**Statistical analysis.** In order to determine the profile of the sample studied, frequency tables and descriptive statistics were employed. The Chi-square test was used to compare categorical variables between the diagnostic groups. The *Kolmogorov-Smirnov* test determined the absence of normal distribution among most of the continuous variables, so non-parametric tests were required. Therefore, when comparing continuous variables between two or three groups, the *Mann-Whitney* U-test and the *Kruskal-Wallis* test were used, respectively. For the *Kruskal-Wallis* test, when p-value<0.05, the comparisons between groups were made using the *Multiple Comparisons Z-score* test.

The data were input to the Epidata software v.3.1. For statistical analysis, the *SPSS* v.17.0 and the *Statistica* v. 7.0 software packages were used. The significance level considered was 5%, i.e. a *p-value*<0.05.

## RESULTS

Table 1 shows the sociodemographic characteristics of participants. It can be noted that the groups were homogeneous with regards to age, education and marital status. There was a significant difference only in family income.

**Table 1:** Sociodemographic profile of participants, stratified by diagnostic group.

Variables		Groups								p-value*
		**NC**			**AD**			**bvFTD**		
		**n=34**	**%**		**n=31**	**%**		**n=31**	**%**	
Gender	Male	21	61.76		17	54.84		19	61.29	
	Female	13	38.24		14	45.16		12	38.71	0.822
Age groups	Average (SD)	65.41	5.88		68.71	6.68		65.61	8.26	0.150
Age of retirement	Average (SD)	59.89	5.90		59.32	5.26		53.53	14.77	0.191
Marital status	Single	1	2.94		0	0.00		1	3.23	
	Married	26	76.47		18	58.06		18	58.06	
	Separated	4	11.76		2	6.45		1	3.23	
	Divorced	2	5.88		7	22.58		2	6.45	
	Widow(er)	1	2.94		3	9.68		7	22.58	
	Stable union	0	0.00		1	3.23		2	6.45	0.087
Education (years of schooling)	Average (SD)	9.56	3.89		8.84	4.61		9.48	5.93	0.623
Family income	Up to 2.0 minimum wages (MW)	1	2.94		15	48.39		15	48.39	
	2.1 to 3.0 times the MW	12	35.29		6	19,35		7	22,58	
	3.1 to 4.0 times the MW	10	29.41		5	16.13		4	12.90	
	More than 4 times the MW	9	26.47		5	16.13		5	16.13	0.003*

* Kruskal-Wallis test followed by multiple comparisons test: Control ≠ AD and Control ≠ bvFTD. NC: normal control; AD: Alzheimer’s disease; bvFTD: behavior variant frontotemporal dementia.

Table 2 shows the clinical characteristics of the sample. On the MMSE, there was a significant difference among the three groups, with the AD group exhibiting the worst performance. The AD group also had a higher number of depressive symptoms than the control group. There were no significant differences between the bvFTD and AD groups when the CDR scores were compared as continuous variables.

**Table 2:** Clinical characteristics of the sample

Variables	Groups								p-value
	**NC**			**AD**			**bvFTD**		
	**Mean**	**SD**		**Mean**	**SD**		**Mean**	**SD**	
CDR	-	-		1.03	0.18		1.23	0.49	0.088
GDS	1.97	0.97		3.45	1.93		2.90	1.96	0.004*
MMSE	25.50	1.31		19.13	2.36		21.90	5.29	<0.001*

* Kruskal-Wallis test. Clinical Dementia Rating scale (CDR), Mini-Mental State Examination (MMSE); Geriatric Depression Scale (GDS). NC: normal control; AD: Alzheimer's disease, bvFTD: behavior variant frontotemporal dementia.

When the CDR was examined as a categorical variable (Table 3), there were a higher number of participants with CDR 0.5 and 2.0 in the group with bvFTD, whereas most of the AD sample had CDR 1.0.

**Table 3:** Disease severity of the clinical groups.

Variables	Groups						p- value
	**NC**	**AD**			**bvFTD**		
		**n**	**Percentage**		**n**	**Percentage**	
CDR 0.5 (very mild)	-	0	0%		2	6.45%	0.004*
CDR 1 (mild)	-	30	96.77%		20	64.52%	
CDR 2 (moderate)	-	1	3.23%		8	25.81%	

* Chi-square test. Clinical Dementia Rating scale (CDR). NC: normal control, AD: Alzheimer's disease, bvFTD: behavior variant frontotemporal dementia.

Table 4 shows that DAFS-BR total and sub-domain scores were significantly lower for patients with bvFTD and AD than for NC, although no differences were noted between the two clinical groups. Regarding the DAD, patients with bvFTD had worse performance than the AD group in Initiation and Planning/Organization, but the two clinical groups were similar in Effective Performance. On the PFAQ, significant differences were found among the three groups, with worst performance reported for the bvFTD group.

**Table 4:** Means and standard deviation for functional performance among the groups.

Functional performance		Groups								p-value
		**NC**			**AD**			**bvFTD**		
		**Mean**	**SD**		**Mean**	**SD**		**Mean**	**SD**	
DAFS-BR	Time orientation	14.82	1.22		11.06	1.88		11.68	2.57	<0.001*
	Communication	12.94	1.37		9.35	1.50		10.13	2.33	<0.001*
	Finance	27.00	3.15		14.68	2.80		16.45	5.67	<0.001*
	Shopping	16.53	2.64		10.16	2.35		12.03	3.42	<0.001*
	Grooming	12.97	0.17		11.90	1.49		11.06	2.11	<0.001*
	Eating	10.00	0.00		9.58	0.67		8.94	1.24	<0.001
	DAFS- Total	93.94	4.30		66.74	7.96		70.29	15.07	<0.001*
DAD	Initiation	-	-		6.65	2.20		4.35	1.60	<0.001**
	Planning and organization	-	-		8.74	1.93		6.00	2.65	<0.001**
	Effective performance	-	-		9.61	2.04		8.45	3.06	0.122
	Total score	-	-		62.42	12.72		46.53	15.73	<0.001**
PFAQ	Total score	0.26	0.57		10.13	5.55		21.23	9.49	<0.001**

* Kruskal-Wallis test followed by multiple comparisons test: Direct Assessment of Functional Status (DAFS-BR); Time Orientation (NC≠bvFTD, NC≠AD); Communication (NC≠bvFTD, NC≠AD); Finance (NC≠bvFTD, NC≠AD); Shopping (NC≠bvFTD, NC≠AD); Grooming (NC≠bvFTD, NC≠AD); Eating (NC≠bvFTD); DAFS - Total Score (NC≠bvFTD, NC≠AD). Disability Assessment for Dementia (DAD), Pfeffer Functional Activities Questionnaire (PFAQ), (NC≠bvFTD, NC≠AD and AD≠bvFTD).

** Mann-Whitney U-test: (AD≠bvFTD); NC: normal control; AD: Alzheimer's disease; bvFTD: behavior variant frontotemporal dementia.

## DISCUSSION

The aim of the present paper was to characterize the functional performance of patients with bvFTD compared to patients with AD and healthy controls. We found that total score, and all DAFS-BR domains, distinguished patients with dementia from healthy individuals. However, the DAFS-BR, as a direct measure of ADLs, did not assist in identifying differences in the functional performance profile of bvFTD and AD patients. The DAD and the PFAQ, both based on caregivers' appraisals, were able to distinguish bvFTD from AD.

It is important to point out the important contribution of instruments that directly assess functionality, such as the DAFS-BR. These instruments do not suffer from potential informant biases and can detect which aspects of functional abilities are most affected at each stage of the disease that may require more caregiver attention. However, there is limited research regarding direct measures of functional abilities in bvFTD. One previous study by Mioshi et. al.^27^ included a performance-based instrument to assess motor (e.g., coordination, grip, transportation) and mental processing skills (e.g., searching, choosing, organizing, sequencing), and their effect on the ability of the person to perform familiar ADL tasks. This study compared bvFTD patients with and without atrophy on neuroimaging exams. Results were inconclusive as there was no correlation between the performance-based scores and scores for the DAD.

As we examined the functional profile generated by the DAFS-BR in the present study, it was noted that patients with bvFTD seemed to display significant impairment in all of the investigated domains, and that the most severely affected were Communication, Shopping, Grooming and Eating skills. Nonetheless, to better characterize functional impairment in bvFTD, DAFS-BR might need to be revised and include other functional domains that rely more significantly on executive functioning and social cognition such as scheduling appointments, planning a trip, or organizing a social event.

Razani et al.^28^ also used the DAFS-R in two previous studies. However, the samples comprised subjects with various sub-types of dementia, including a small number of patients with bvFTD. Therefore, it was not possible to examine the functional profile of each form of dementia separately. In the first study,^28^ the authors aimed at verifying the correlation between performance in executive function tasks and direct functional performance among patients with dementia. The results suggested that lower scores on the DAFS-R were associated with poor executive performance. In the study by Razani et al.,^29^ impairment in the IADL sub-domains of the DAFS-R (Time Orientation, Communication and Finances) were associated with greater burden to caregivers.

In the current study, for the DAD, the bvFTD group exhibited worse performance than AD patients on the domains of Initiation and Planning/Organization, and on total score. It is noteworthy that Effective performance was comparable between the two clinical groups. These findings are in line with those reported by Mioshi et al.^9^ that the DAD was able to detect differences in the functional performance of patients with bvFTD compared to patients with SD, NFPA or AD. Mioshi et al.^9^ and Kipps et al.^10^ reported that impairments in ADLs among patients with bvFTD were more severe than those displayed by patients with AD. In Kipps et al.,^10^ however, there were significant differences between bvFTD and AD in DAD total score and Effective performance. Differences on the DAD sub-domains between the latter and the current study may be due to differences in sample size.

In contrast with the above-mentioned findings, Bahia et al.^23^ failed to find any significant differences between patients with AD and FTLD for DAD scores. One possible explanation for this discrepancy is related to methodological differences, such as the fact that the FTLD group was not divided into dementia sub-types, therefore, the study did not allow a direct comparison between bvFTD and AD.

On the PFAQ, patients with bvFTD had higher scores, suggesting that they have a greater degree of dependency to perform IADLs compared to individuals with AD. It should be pointed out that the PFAQ was able to distinguish bvFTD from AD patients, in contrast with the observed results from the DAFS-BR. It is possible that this difference stems from the fact that the PFAQ examines the degree of patient dependency or that research centers may have relied more significantly on the PFAQ to classify patients into diagnostic groups.

Regarding possible explanations for the severe functional impairment documented in bvFTD, researchers have considered several explanations. Authors such as Mioshi et al.^27^ have suggested that besides impairment in executive functions, neuropsychiatric symptoms such as apathy and impulsiveness, frequently observed among this patient group, may also influence functional performance. Studies involving scales of severity and staging of bvFTD, such as the investigations by Marra et al.,^30^ Mioshi et al.^27^ and Josephs et al.,^1^ support the hypothesis that faster disease progression, together with its cognitive and neuropsychiatric symptoms, may modulate functional performance.

The present study highlighted the importance of carrying out functional assessment of patients with suspected bvFTD, given the relevance of these changes for the diagnosis and clinical management of this dementia sub-type. One limitation of the study relates to sample size as it may have hindered the identification of small group differences. In addition, there were a higher number of individuals with CDR 2 in the bvFTD group (although diagnostic groups were similar for CDR when analyzed as a continuous variable) and this fact may have exacerbated some of the group differences.

In conclusion, the results from the present research study corroborate that individuals with bvFTD display greater functional impairment compared to individuals with AD. Findings also suggest that direct and indirect assessments provide relevant information about the functional status of the patient. In the current study however, caregiver based instruments were more useful for detecting nuances in the functional profile of the clinical groups. Further studies investigating the clinical characterization of bvFTD are needed. Research studies with larger samples, examining the association between functional performance and caregiver burden are

recommended.

## Figures and Tables

**Figure 1:**
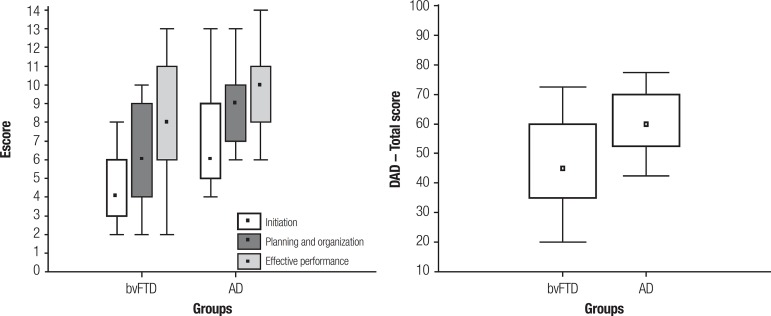
Initiation, planning and organization, effective performance, total score, disability assessment for dementia (DAD).
